# A Possible Method for Non-Hermitian and Non-*PT*-Symmetric Hamiltonian Systems

**DOI:** 10.1371/journal.pone.0097107

**Published:** 2014-06-04

**Authors:** Jun-Qing Li, Yan-Gang Miao, Zhao Xue

**Affiliations:** 1 School of Physics, Nankai University, Tianjin, China; 2 Kavli Institute for Theoretical Physics China, Chinese Academy of Sciences, Beijing, China; 3 Bethe Center for Theoretical Physics and Institute of Physics, University of Bonn, Bonn, Germany; University of Nottingham, United Kingdom

## Abstract

A possible method to investigate non-Hermitian Hamiltonians is suggested through finding a Hermitian operator *η*_+_ and defining the annihilation and creation operators to be *η*_+_ -pseudo-Hermitian adjoint to each other. The operator *η*_+_ represents the *η*_+_ -pseudo-Hermiticity of Hamiltonians. As an example, a non-Hermitian and non-*PT*-symmetric Hamiltonian with imaginary linear coordinate and linear momentum terms is constructed and analyzed in detail. The operator *η*_+_ is found, based on which, a real spectrum and a positive-definite inner product, together with the probability explanation of wave functions, the orthogonality of eigenstates, and the unitarity of time evolution, are obtained for the non-Hermitian and non-*PT*-symmetric Hamiltonian. Moreover, this Hamiltonian turns out to be coupled when it is extended to the canonical noncommutative space with noncommutative spatial coordinate operators and noncommutative momentum operators as well. Our method is applicable to the coupled Hamiltonian. Then the first and second order noncommutative corrections of energy levels are calculated, and in particular the reality of energy spectra, the positive-definiteness of inner products, and the related properties (the probability explanation of wave functions, the orthogonality of eigenstates, and the unitarity of time evolution) are found not to be altered by the noncommutativity.

## Introduction

A non-Hermitian Hamiltonian with a complex potential usually has complex eigenvalues and such a system does not maintain the conservation of probability. However, the non-Hermitian Hamiltonian with a class of quasi-Hermiticity was proposed [1]–[4] in which the real eigenvalues and the conservation of probability are possible. Recently the real eigenvalues and corresponding eigenstates of a non-Hermitian Hamiltonian associated with some symmetry have been paid more attention to, such as the 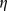-pseudo-Hermitian Hamiltonian [5]–[8] and the 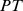-symmetric Hamiltonian [9], [10]. The former satisfies
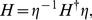
(1)where the invertible operator 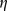 is linear Hermitian, and the latter satisfies the 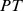 symmetry, 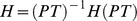, where 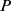 and 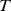 stand for the parity and time reversal transformations, respectively. In addition, some experiments [11], [12] on 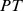-symmetric (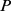-pseudo-Hermitian) Hamiltonians have been carried out in the region of optics.

Roughly speaking, there exist two methods that are used to study an ordinary (Hermitian) Hamiltonian system. The usual one focuses on solving the Schrödinger equation under certain boundary conditions in order to calculate eigenvalues and eigenstates. The other method, which is useful for dealing with the systems such as the harmonic oscillator, is associated closely with annihilation and creation operators and their commutation relations. Nevertheless, in quantum theories on non-Hermitian Hamiltonians, the former method is commonly adopted in literature, see, for instance, the review article [13], while the latter cannot be utilized directly because imaginary terms appear in a non-Hermitian Hamiltonian. In this paper we give a possible method through redefining annihilation and creation operators, and as an application beyond the non-Hermitian 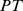-symmetric quantum theory [9], [10], we at first construct a non-Hermitian and non-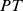-symmetric Hamiltonian that is decoupled, analyze its spectrum and inner product, and then apply our method to a coupled Hamiltonian that is given by extending the decoupled one to the canonical noncommutative space. Our method is therefore complementary to the non-Hermitian 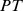-symmetric method.

This paper is organized as follows. In the next section entitled **The method for 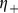-pseudo-Hermitian systems**, we elaborate our method for an 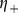-pseudo-Hermitian Hamiltonian by redefining annihilation and creation operators that are 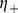-pseudo-Hermitian (no longer Hermitian) adjoint to each other. In general, 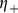 does not coincide with 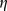. The subscript “+” means that 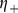 is associated with a positive-definite inner product. The key point of this method is to find out an 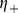 operator that represents an inherent symmetry of the non-Hermitian Hamiltonian, *i.e.*, the 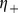-pseudo-Hermiticity, also called the 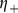-pseudo-Hermitian self-adjoint condition. With such an 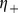, one can deduce that the non-Hermitian Hamiltonian has a real spectrum with lower boundedness and a positive-definite inner product. Here we have to mention an earlier work [14] which also dealt with a non-Hermitian Hamiltonian system. Although the real spectrum was given there, the non-Hermiticity was not properly treated and more severely the positive-definiteness of inner products was completely ignored. In fact, the annihilation and creation operators defined in ref. [14] are no longer Hermitian adjoint to each other, which gives rise to the incorrect treatment to that non-Hermitian Hamiltonian. In the section entitled **The non-Hermitian and non-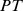-symmetric system**, as an application beyond the non-Hermitian 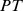-symmetric quantum theory [9], [10], we construct a non-Hermitian and non-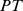-symmetric Hamiltonian that is decoupled, and find out 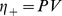 through introducing a special operator 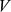. Then we redefine the annihilation and creation operators in such a way that they are 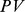-pseudo-Hermitian adjoint to each other, and as expected, obtain a real energy spectrum and a positive-definite inner product. In the section entitled **Noncommutative extension**, the decoupled non-Hermitian and non-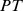-symmetric Hamiltonian is extended to the canonical noncommutative phase space with noncommutative coordinate operators and noncommutative momentum operators as well, and it turns out to be coupled. Our method is applicable to the coupled Hamiltonian. We then calculate the noncommutative corrections of energy levels up to the first and second orders in noncommutative parameters, respectively, and in particular we give an interesting result that the reality of energy spectra and the positive-definiteness of inner products are not altered by the noncommutativity of phase space. Finally, we make a conclusion in the section of the title **Conclusion**.

## The Method for 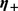-Pseudo-Hermitian Systems

At first, we review the modified inner product. The condition that an 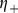-pseudo-Hermitian observable 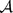 should obey takes the following form [5], [8], [15], *i.e.*, the 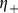-pseudo-Hermitian self-adjoint condition,
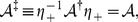
(2)where the superscript "
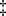
" stands for the action of an 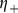-pseudo-Hermitian adjoint to an operator. Note that the 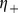-pseudo-Hermitian adjoint becomes the ordinary Hermitian adjoint when 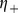 takes the identity operator, in which case the 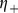-pseudo-Hermitian Hamiltonian turns back to the Hermitian Hamiltonian. Related to eq. (2), the definition of the modified bra vector states is as follows:
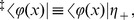
(3)where 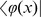 denotes a usual bra vector state that is Hermitian adjoint to a (usual) ket vector state 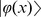, *i.e.*, 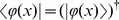. It is convenient to use the notation of the left hand side of eq. (3), *i.e.*, the notation with hidden 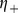, which will be seen evidently in the following context. The modified bra vector state may be called the 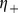-pseudo-Hermitian adjoint to the ket vector state and it becomes the normal one in the Hermitian quantum mechanics where 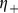 is just the identity operator. Therefore, the modified inner product in the Hilbert space for an 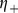-pseudo-Hermitian Hamiltonian system naturally has the form,
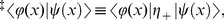
(4)which can be understood as a generalized inner product. Since 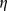 (see eq. (1)) was called by Pauli [5] the indefinite metric in the Hilbert space, 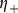 is then called the positive-definite metric because it gives rise to [1]–[4], [16] a real and positive-definite norm or probability, 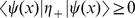. Note that the operator 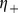 is in general required [5]–[8] to be linear Hermitian and invertible, which ensures not only the reality of the average of physical observables but also the reality and positivity of the probability. (In our recent work [16] we discuss the anti-linear anti-Hermitian case and obtain some interesting results.) In addition, we mention that the self-adjoint condition (eq. (2)) is consistent with the modified inner product (see eq. (4)), *i.e.*, 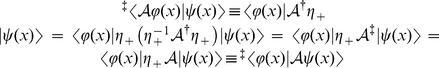. We point out that it is the requirement of positive norms that makes it a quite nontrivial task to find out the metric 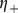 even for a simple non-Hermitian Hamiltonian, which can be seen clearly from our non-Hermitian and non-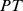-symmetric models in the two sections below.

Next, we redefine the creation operator as the 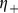-pseudo-Hermitian adjoint to a particularly chosen annihilation operator (for concrete procedures see the following two sections) as follows:
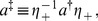
(5)which is quite different from the definition in the Hermitian quantum mechanics. Note that the redefined creation and annihilation operators are 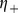-pseudo-Hermitian adjoint to each other, that is, we have 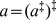. It is easy to verify this equality, that is, 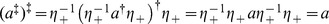, where the Hermiticity of 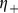 has been utilized. In addition, we can prove that 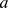 and 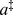 are 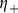-pseudo-Hermitian adjoint to each other with respect to the generalized inner product eq. (4), that is, considering eqs. (4) and (5) we get
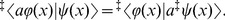
(6)


We can verify 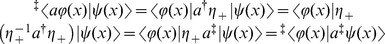. Eq. (6) shows that the redefinition of the annihilation and creation operators is consistent with the definition of the modified bra vector states eq. (3). The formula 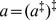 becomes the one we are quite familiar with, *i.e.*, 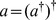, when 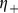 takes the identity operator, *i.e.*, when an 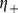-pseudo-Hermitian system becomes a Hermitian one. Considering the well-known commutation relations satisfied by the usual annihilation and creation operators in the conventional quantum mechanics, we require that the redefined annihilation and creation operators in the 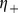-pseudo-Hermitian quantum mechanics comply with
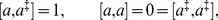
(7)which turn s consistently back to the usual commutation relations when 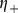 becomes the identity operator, *i.e.*, that 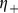 becomes the identity operator is equivalent to that the 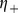-pseudo-Hermitian self-adjoint becomes the Hermitian self-adjoint, 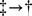.

At last, we define the corresponding number operator in the pseudo-Hermitian quantum mechanics as follows:
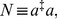
(8)which, as a physical observable, is of course 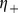-pseudo-Hermitian self-adjoint, *i.e.*, 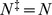. (Considering eqs. (5) and (8), we have 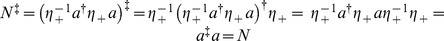, where the Hermiticity of 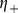 has been used.) More precisely, the number operator 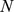 is self-adjoint with respect to the generalized inner product,
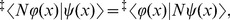
(9)which can be proved in this way: in terms of eq. (4) we derive 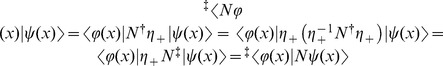. Eq. (9) shows that the definition of modified bra and ket vector states is consistent with the self-adjoint requirement of physical observables. Using eqs. (7) and (8), we can verify the following commutation relations in the pseudo-Hermitian quantum mechanics,



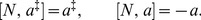
(10)Consequenly, we provide a possible method for an 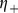-pseudo-Hermitian system. The remaining task is just to deduce some useful formulae, such as the 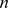-particle state and the ladder property of redefined creation and annihilation operators, which will be fulfilled at the end of this section for the completeness of our method.

In addition, we note that the unitarity of time evolution is guaranteed with respect to the modified inner product (eq. (4)) in the 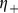-pseudo-Hermitian quantum mechanics. Considering the 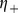-pseudo-Hermitian self-adjoint of the Hamiltonian, *i.e.*, 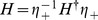, and the time evolution of an initial state 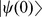, 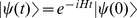, we have
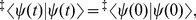
(11)which gives the unitary time evolution. It is obvious to prove the relation: 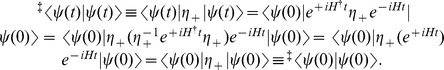


As a summary, we point out that the characteristic of our method is to adopt the orthonormal basis of Hamiltonians. Although the biorthonormal basis [17], [18] has been applied [6]–[8] to pseudo-Hermitian Hamiltonian systems, it is interesting to investigate whether the usual treatment (to consider just the orthonormal basis of Hamiltonians but not that of the Hermitian conjugate of Hamiltonians) is still available to such non-Hermitian systems. Our way to realize this goal is to find out the specific operator 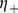 and then to make the redefinition of annihilation and creation operators (eq. (5)) that are adjoint to each other with respect to the generalized inner product (eq. (4)). For the concrete procedure, see the next two sections. We emphasize that the operator 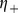 is in general nontrivial, that is, it is impossible to reduce 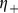 to be the identity through a basis transformation. The reason is that the Hamiltonian of an 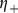-pseudo-Hermitian system is no longer Hermitian self-adjoint with respect to the usual inner product: 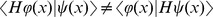, but 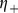-pseudo-Hermitian self-adjoint with respect to the generalized inner product (see eqs. (3) and (4)): 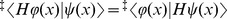. The operator 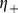, as an inherent symmetry of 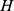, can never be eliminated through a basis transformations. Finally, we make a comment that our method may be understood as a generalized Fock space representation for non-Hermitian and non-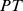-symmetric quantum systems due to the existence of a nontrivial 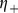, and that it can be applied to deal with such systems mainly by the redefinitions of annihilation, creation, and number operators and by the reconstruction of their commutation relations.

Now we fulfill the derivation of some useful formulae by using our specific notation of the modified bra vector state (see eq. (3)). It is obvious that this process reduces apparently to that of the ordinary (Hermitian) quantum mechanics when 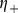 becomes the trivial identity operator.

Let us at first derive the 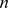-particle state. If 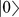 stands for the ground state and 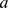 annihilates the ground state, 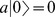, we calculate the average value of 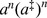 with respect to the ground state and its 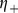-pseudo-Hermitian adjoint (the modified bra vector state, see eq. (3)) by repeatedly using eq. (7),
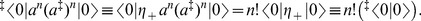
(12)If 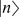 is defined by
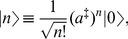
(13)we obtain its 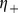-pseudo-Hermitian adjoint by using eqs. (3) and (5),
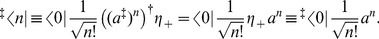
(14)Therefore, we can rewrite eq. (12) using the notation with hidden 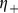 as
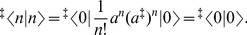
(15)Moreover, by using eqs. (7), (8) and (13) and considering 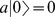 again, we derive
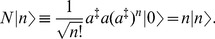
(16)Combining eq. (15) and eq. (16), we obtain
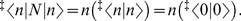
(17)Consequently, if the ground state is normalized with respect to the generalized inner product (eq. (4)), *i.e.*, 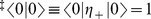, the state defined by eq. (13) is convinced to be the expected 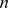-particle state and the operator 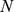 defined by eq. (8) is then confirmed to be the desired number operator. Moreover, we can deduce the orthogonality of eigenstates, 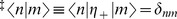. In the sections **The non-Hermitian and non-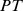-symmetric system** and **Noncommutative extension**, for instance, the exact forms of 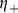 operators are provided for the concrete models, the ground state can be determined to be normalized, and the orthogonality of eigenstates is guaranteed.

Now we calculate the ladder property of redefined creation and annihilation operators. It is straightforward from eq. (13) to have
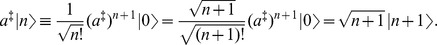
Multiplying the above equation by the operator 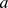 from the left and using eqs. (7), (8) and (16), we get
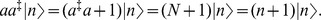
Combining the above two equations, we obtain 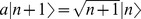. As a result, we give the following ladder properties for the operators 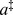 and 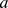, respectively,
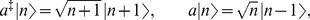
(18)which indeed shows that 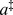 has the function of creation and 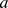 that of annihilation as expected.

## The Non-Hermitian and Non-*PT*-Symmetric System

In this section we investigate a concrete non-Hermitian Hamiltonian by means of the method provided in the above section. In order to show that our method is complementary to the non-Hermitian 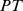-symmetric method [9], [10], we construct a non-Hermitian and non-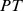-symmetric Hamiltonian. We add two imaginary terms which are proportional to 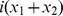 and 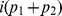, respectively, to the Hamiltonian of an isotropic planar oscillator, and then give a new Hamiltonian:
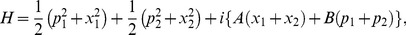
(19)where 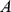 and 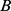 are real parameters; 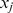 and 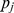 (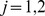) are two pairs of canonical coordinate operators and their conjugate momentum operators, they are Hermitian and satisfy the standard Heisenberg commutation relations, where 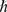 is set be unity through out this paper. This Hamiltonian is decoupled, and normally it is enough for us to analyze its one-dimensional part in this section. However, we shall see that this is a good enough model for us to illustrate our method clearly, and that it is quite nontrivial to find out operator 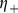 for such a simple model. In particular, the decoupled Hamiltonian will turn out to be coupled when it is extended to the noncommutative space in the next section, and our method is still applicable to the coupled Hamiltonian. That is the reason why we choose such a decoupled two-dimensional Hamiltonian in this section.

The Hamiltonian eq. (19) is obviously non-Hermitian and non-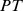-symmetric due to the different properties of 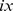 and 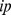 under the 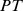 transformation [9], [10]. For a non-Hermitian *but* 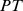-symmetric Hamiltonian, there exists a well-established theory called 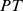-symmetric quantum mechanics [13]. In order to extend our discussion beyond the 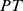-symmetric theory, we particularly construct the non-Hermitian and non-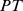-symmetric Hamiltonian depicted by eq. (19), convert it into an 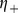-pseudo-Hermitian Hamiltonian, and then deal with it by using the method demonstrated in the above section.

We notice that the decoupled Hamiltonian can easily be diagonalized and rewritten as
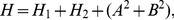
(20)where the new variables are defined by
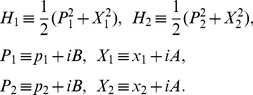
(21)Eq. (20), together with eq. (21), looks like the usual Hamiltonian of a decoupled two-dimensional harmonic oscillator, but in fact, it is not the case. Now 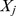 and 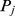, though satisfying the standard Heisenberg commutation relations,
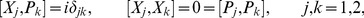
(22)are not Hermitian self-adjoint, but as expected, they are 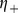-pseudo-Hermitian self-adjoint and have real average values with respect to the generalized inner product (cf. eq. (4)), and so are 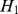 and 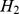.

Now we begin the investigation of the system governed by the Hamiltonian eq. (19) or eq. (20). It is subtle to find 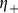 for the system.

We give a two-step way for the construction of the desired operator 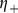. In the first step, inspired by Lee and Wick [19], we define operator 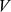 as follows,



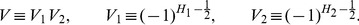
(23)

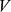
, 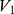, and 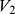 are invertible. Note that the exponential factor 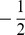 in 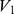 and 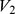 is introduced in order to eliminate the zero-point energy in 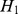 and 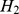 and then to express 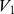 and 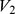 in terms of number operators (cf. eq. (38)). Here 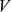 is 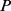-pseudo-Hermitian self-adjoint (see the proof at the end of this section),
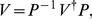
(24)which is different from the case in ref. [19] where a Hermitian operator was introduced. Moreover, we point out that 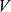 is defined in terms of the Hamiltonian, which is more intuitive than the definition of the operator 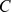 [9], [10] for positive-definite inner products in the 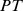-symmetric quantum mechanics, where 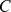 is defined by unknown eigenstates of a non-Hermitian *but* 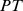-symmetric Hamiltonian. Then, in the second step we set 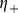 be the product of 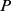 and 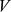,
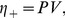
(25)which is linear Hermitian and invertible. Note that 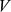 is linear non-Hermitian because 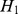 and 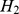 are non-Hermitian. It is easy to prove the Hermiticity of 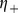 by considering the 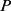-pseudo-Hermitian self-adjoint of 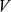 (eq. (24)), that is, 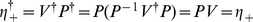. In addition, because 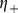 is related to the Hamiltonian through 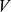 the generalized inner product with respect to this 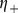 (see eq. (4)) can be called a dynamical inner product as the 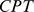 inner product [9], [10] was called.

Using eqs. (21)–(23), we deduce the 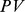-pseudo-Hermiticity of 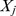 and 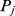,
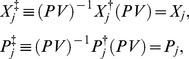
(26)which can be proved as follows.

We just verify the case 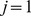, *i.e.*, 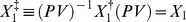 and 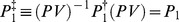. As to 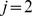, the procedure is exactly the same.

Starting from
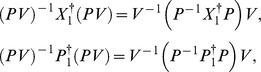
we at first get 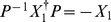 and 
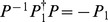
 in terms of eq. (21). Then, considering eq. (22) and eq. (23) we obtain
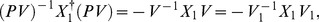
(27)

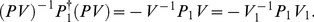
(28)


When applying the BCH formula,
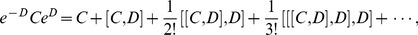
(29)to eq. (27), *i.e.*, letting 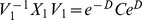, we have the corresponding operators 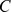 and 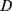 with eq. (21) and eq. (23),



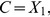
(30)

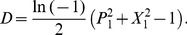
(31)


Thus, we compute the commutation relations by using eq. (22),
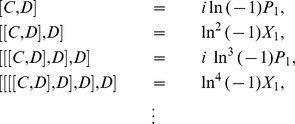
and acquire
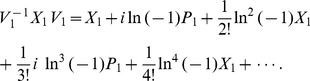
(32)Further considering.
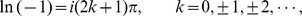
we have
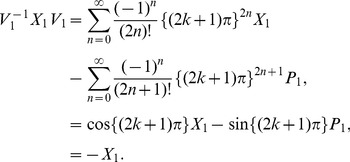
(33)Combining eq. (27) with eq. (33), we reach our goal,
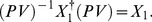
(34)In addition, when we apply the BCH formula to eq. (28), *i.e.*, let 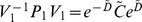, the corresponding operator 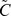 takes the form,
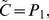
(35)and 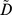 is same as 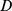 (see eq. (31)). Therefore, from the commutation relations,
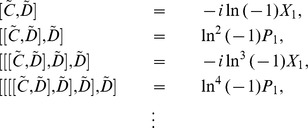
we obtain
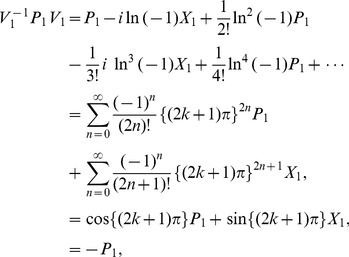
(36)and thus verify
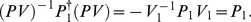
(37)This ends the proof of eq. (26).

If 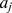 is specifically chosen as 
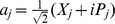
, 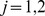, we have from eq. (5) the operator 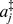 as the 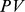-pseudo-Hermitian adjoint to the operator 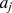, 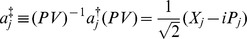, 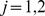. This shows the subtleness of the construction of 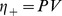 because we can then rewrite the Hamiltonian eq. (19) in terms of 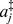 and 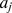 that can be verified to satisfy the basic requirement in terms of eq. (22), 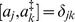, 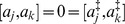, 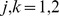. In accordance with the formulae given in the above section, we can now write the number operator which is 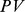-pseudo-Hermitian self-adjoint, 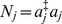, 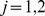, where repeated subscripts do not sum except for extra indications, and get the expected commutation relations by using the algebraic relations of 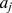 and 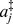 and the expression of the number operator, 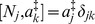, 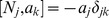, 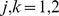. Furthermore, given 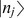 a set of eigenstates of the number operator 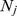, *i.e.*, 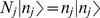, 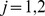, if its inner product defined by eq. (4) is positive definite, 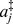 and 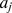 can finally be convinced to be the creation and annihilation operators that satisfy the property of ladder operators, 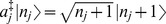, 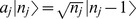, 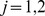, and 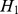 and 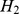 can be expressed in terms of the number operators as follows:
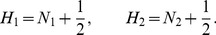
(38)


**Figure 1:**
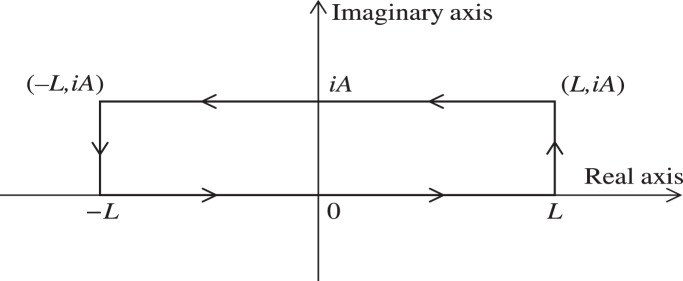
We choose the rectangle with length *L* and width *A* in the complex plane as the contour.

At present we turn to the verification that the generalized inner product defined by eq. (4) is positive definite for our choice 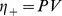. Due to eq. (17), we only need to prove the normalization of the ground state, 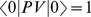. Utilizing eqs. (23) and (38) together with the property of the number operator, we have 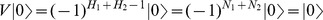, and thus obtain 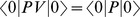. In addition, considering the wavefunction of the ground state, 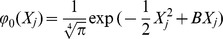, where 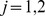, together with the definitions of 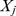 and 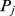 (see eq. (21)), we can calculate the generalized inner product with respect to the ground state in terms of the Cauchy’s residue theorem of the complex function theory (see Figure 1 for the details),
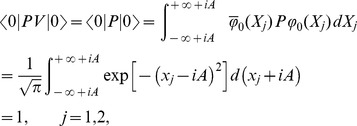
(39)where 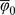 denotes the complex conjugate to 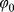. Note that the symbols 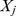 and 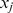 in the above equation no longer stand for operators but coordinates. Eq. (39) definitely gives the normalization of the ground state, which, together with eq. (17), leads to 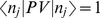, where 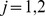. That is, we at last prove the positive definiteness of the generalized inner product (defined by eq. (4)) for the set of eigenstates of the number operator, 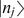.

Alternatively, we can exactly solve the wavefunctions for any excited states related with the Hamiltonian eq. (19) or eq. (20):
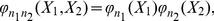
(40)where
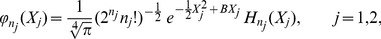
(41)and 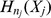 denotes the Hermite polynomial of the 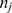-th degree with the argument 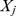. Therefore, by considering 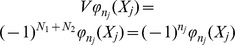 and using the same contour as in Figure 1 we can prove that the inner product for oscillator 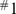 or oscillator 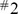 is orthogonal and normalized, *i.e.*,
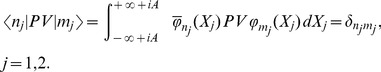
(42)This shows from an alternative point of view that the positive definiteness of the inner product is guaranteed.

As a result, using eq. (38) we can easily rewrite the Hamiltonian eq. (20) in terms of the number operators as follows:
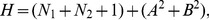
(43)and then give its real and positive spectrum,
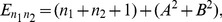
(44)where 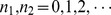. In an alternative way, we can get the same spectrum eq. (44) if the Hamiltonian eq. (43) acts directly on the eigenfunction eq. (40).

In addition, we point out that the non-Hermitian and non-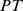-symmetric Hamiltonian (eq. (19)) has the inherent 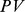-pseudo-Hermiticity,
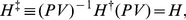
(45)which was unknown initially *but* is exposed later by the finding of the operator 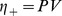. This property is quite obvious when we verify it by using eqs. (20)–(23), that is, 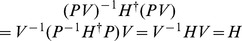, where 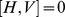 is used in the last equality. Moreover, we emphasize that 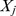 and 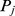 also have 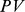-pseudo-Hermiticity, see eq. (26), and thus they, rather than the Hermitian operators 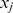 and 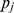, are physical observables with real average values under the generalized definition of inner products (cf. eq. (4)). We conclude that this 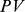-pseudo-Hermitian Hamiltonian, though non-Hermitian and non-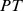-symmetric, possesses a real spectrum with lower boundedness and a positive-definite inner product, and that it can be understood as a generalized harmonic oscillator in the sense of 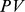-pseudo-Hermitian quantum theory.

At the end of this section we perform the verification of eq. (24). Using eq. (23) and the Taylor expansion, we have
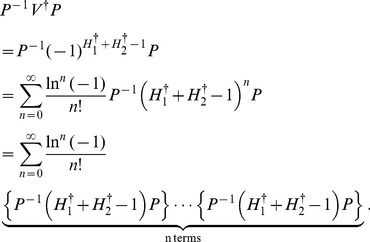
(46)Considering eq. (21), we get
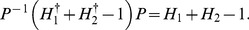
(47)Substituting eq. (47) into eq. (46), we thus obtain
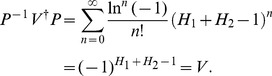
(48)


## Noncommutative Extension

In the 1930s, Heisenberg [20] proposed a kind of lattice structures of spacetimes, *i.e.*, the quantized spacetime now called the noncommutative spacetime, in order to overcome the ultraviolet divergence in quantum field theory. Later Snyder [21], [22] applied the idea of spacetime noncommutativity to construct the Lorentz invariant field theory with a small length scale cut-off. Since the Seiberg-Witten's seminal work [23] on describing some low-energy effective theory of open strings by means of a noncommutative gauge theory, the physics founded on noncommutative spacetimes has been studied intensively, see, for instance, some review articles [24], [25]. As a result, it is quite natural to ask how an 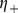-pseudo-Hermitian Hamiltonian behaves on a noncommutative space. That is, it is interesting to investigate whether the 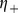-pseudo-Hermitian symmetry, the real spectrum and the positive-definite inner product of a non-Hermitian and non-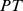-symmetric system maintain or not when the system is extened to a noncommutative space. Incidentally, one of the authors of the present paper established [26] a noncommutative theory of chiral bosons and found that the self-duality that exists in the usual chiral bosons is broken in the noncommutative chiral bosons.

We consider a general two-dimensional canonical noncommutative space with noncommutative spatial coordinate operators and noncommutative momentum operators as well,
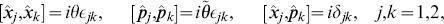
(49)where 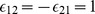, and 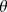 and 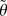 independent of coordinate and momentum operators are real noncommutative parameters which are much smaller than the Planck constant. Therefore, we extend our system (eq. (19)) to this noncommutative space in a straightforward way,
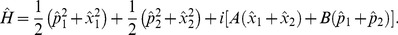
(50)In accordance with the commutation relations in the two spaces, *i.e.*, the standard Heisenberg commutation relations and eq. (49), we establish the following relationship between the commutative and noncommutative spaces up to the first order in 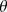 and 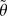,
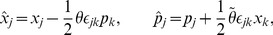
(51)where the repeated subscripts mean summation, and then rewrite eq. (50) in terms of the coordinate and momentum operators of the commutative space still up to the first order in 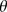 and 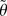,
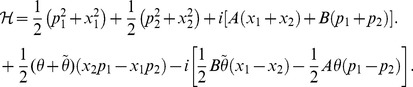
(52)The last two terms in the above Hamiltonian give the noncommutative corrections, where the first that presents the coupling of the two one-dimensional oscillators is Hermitian while the second is not. Note that this Hamiltonian is still non-Hermitian and non-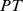-symmetric, and that it is also coupled and non-diagonalized. In addition, we point out that eq. (50) is symmetric under the permutation of dimension 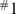 and dimension 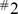 while eq. (52) does not possess such a permutation symmetry because the relationship between the commutative and noncommutative spaces (eq. (51)) breaks this symmetry under the first order approximation to the noncommutative parameters.

After analyzing eq. (52), we partially diagonalize it up to the first order in 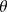 and 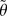,
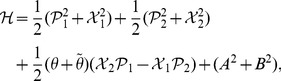
(53)where the new variables are defined as follows:
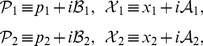
(54)and new real parameters 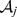 and 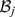, 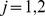, are defined by
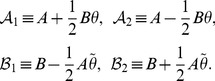
(55)The new variables, 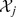 and 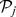, where 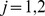, are non-Hermitian like that in the commutative case (see eq. (21)), and satisfy the same Heisenberg commutation relations as eq. (22), which is crucial for us to apply our method to the noncommutative extension.

We note that the third term in eq. (53) gives the first order correction in the noncommutative parameters. This term describes the coupling of oscillator 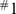 and oscillator 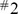 and thus needs to be dealt with particularly because no couplings exist in the commutative case.

Following the procedure stated in the above section for searching for the operator 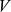, we at first find out the corresponding operator 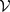 for the noncommutative case, 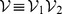, where 
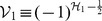
 and 
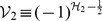
, and 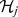
's are defined as 
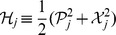
, 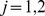. Note that 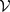 is linear and invertible, but non-Hermitian, and it is also 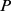-pseudo-Hermitian self-adjoint as 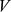, *i.e.*, 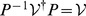, whose verification is similar to eq. (24). Then we give the expected operator 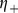 as 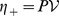, which can be proved to be Hermitian though 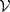 is not.

Although it is not easy to set a suitable 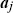 because the coupling term appears in the noncommutative case (see eq. (53)), we find out the desired 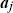,
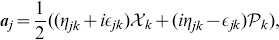
(56)where the repeated subscripts denote summation, and 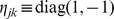. By considering the 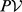-pseudo-Hermiticity of 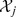 and 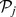, which is same as that in the commutative case (see eq. (26)), we therefore obtain from eq. (5) the 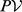-pseudo-Hermitian adjoint to 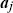,
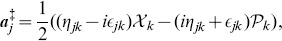
(57)where the repeated subscripts denote summation. We can show that the algebraic relations of 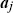 and 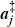 are same as that of the commutative case, which is the result we expect to. Further, we give the number operator which is 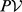-pseudo-Hermitian self-adjoint, 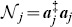, 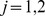, and find that 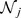, 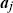 and 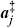 have the same commutation relations as that of the commutative case. Similarly, for a given set of eigenstates of the number operator 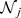, *i.e.*, 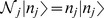, we can prove (see below) that 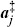 and 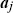 are indeed the creation and annihilation operators we are looking for, that is, they satisfy the property of ladder operators.

Now we can write the Hamiltonian eq. (53) in a completely diagonalized form by means of the number operator 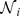,
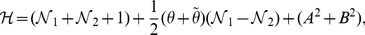
(58)and easily give the real and positive energy spectrum up to the first order in the noncommutative parameters,
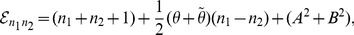
(59)where 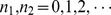. Note that the first order correction of the spectrum is proportional to the difference between the eigenvalue of oscillator 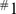 and that of oscillator 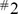. We point out that the first order correction of the energy spectrum is vanishing when the noncommutative parameters satisfy the special relation 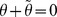, in which case higher order corrections might be considered. Moreover, if 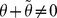 but 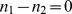, *i.e.*, the energy eigenvalues of oscillator 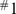 and oscillator 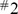 equal, there is no first order correction for the spectrum, either. For instance, it is obvious that the energy level of the ground state is not modified because of 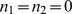. However, we emphasize that the noncommutative corrections of the eigenfunction are non-vanishing even for the two cases (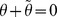, and 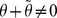 but 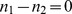) because the eigenfunction, as stated in the above section, has the same formulation (see the next paragraph for a detailed analysis) as eqs. (40) and (41) with the replacement of 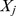 by the new coordinates 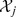 (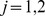) given in eq. (54), and thus contains the noncommutative parameter 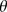 through 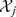. This would be seen more evidently from eq. (58) which is the diagonalized form of eq. (53).

We turn to the proof of the positive-definite inner product in the noncommutative case, which shows as in the commutative case that 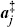 and 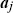 are the creation and annihilation operators that satisfy the property of ladder operators. Because the coupling part is commutative with the free part in the Hamiltonian eq. (53), that is,
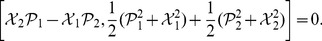
(60)


Such a commutativity can be seen more clearly from eq. (58), *i.e.*, 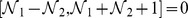. We conclude that the eigenfunction of the total Hamiltonian (eq. (53)) is the product of the eigenfunctions of the oscillator 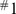 Hamiltonian and oscillator 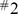 Hamiltonian. As a result, it takes the same form as that obtained in the above section just with the replacement of 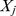 by 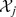, where 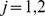. For example, the eigenfunction of the ground state for one of the oscillators is: 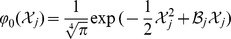, where 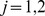, and repeated subscripts do not sum. Similar to the commutative case in the section **The non-Hermitian and non-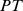-symmetric system** (see eq. (39)), by using 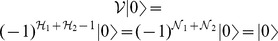, we have 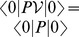, and can therefore prove the normalization of the ground state under the generalized inner product in terms of the Cauchy's residue theorem together with the contour chosen in Figure 1, *i.e.*, 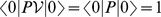. Moreover, we can also prove the orthogonality and normalization of the inner products of excited states, like eq. (42) for the noncommutative case. This completes the proof of the positive definiteness of the generalized inner product defined by eq. (4) with 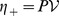.

As analyzed in the section **The non-Hermitian and non-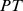-symmetric system** for the commutative case, we can verify straightforwardly from eq. (58) that the Hamiltonian inherently has the 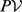-pseudo-Hermiticity in the noncommutative case,
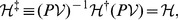
(61)which was unknown before the finding of 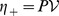. As a consequence, in the noncommutative generalization we confirm that the reality of energy spectra with lower boundedness and the positive definiteness of inner products maintain because the 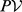-pseudo-Hermitian symmetry exists in the system depicted by the Hamiltonian eq. (52), or eq. (53), or eq. (58). In addition, besides this Hamiltonian, the other physical observables whose average values are real also have 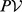-pseudo-Hermiticity, such as the coordinate 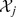 and the momentum 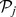.

Following the above treatment for investigating the effect of the first order correction on the noncommutative model, we can calculate the higher order corrections, such as the second order correction. We shall see an interesting property that the second order correction of the Hamiltonian is decoupled and thus the treatment for the first order correction is still applicable to the second order case. The details are given below.

At first we propose the following relationship between the commutative and noncommutative spaces up to the second order in 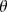 and 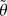,
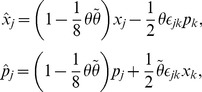
(62)where the repeated subscripts mean summation. Substituting the above relations into eq. (50), we obtain the Hamiltonian in terms of the coordinate and momentum operators of the commutative space still up to the second order in 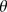 and 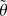,
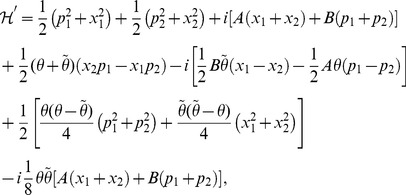
(63)where the first two lines of the above equation exactly cover eq. (52), *i.e.*, the Hamiltonian up to the first order in 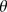 and 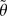. One can see that the last two lines give the second order correction and they are decoupled.

Then, following the treatment given for 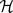 (eq. (52)), we partially diagonalize 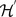 up to the second order in 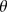 and 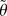,
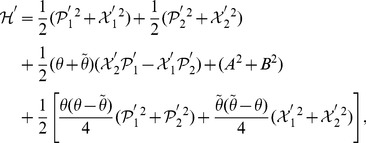
(64)where the new symbols are defined as follows:
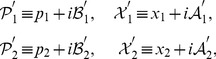
(65)and
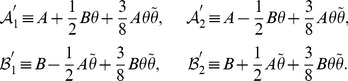
(66)


Next, considering the second order correction in eq. (64) we find the corresponding annihilation and creation operators,
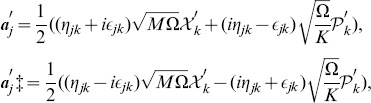
(67)where the new parameters are defined by
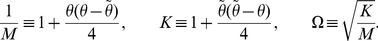
(68)After introducing the number operator 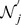 associated with 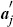 and 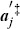 as 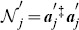, where 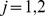, we can rewrite the Hamiltonian 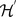 in a completely diagonalized form,
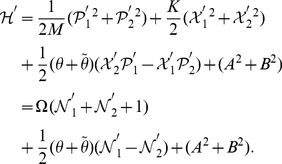
(69) As a result, the energy spectrum up to the second order in 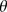 and 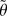 reads
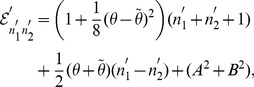
(70)where 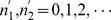.

At last, we note that 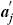 and 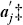 are 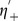-pseudo-Hermitian adjoint to each other, and 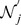 is 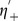-pseudo-Hermitian self-adjoint, where 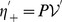. Similar to the first order case, here 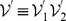, 
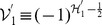
 and 
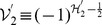
, and in particular, 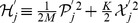, where 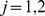. The other related properties can be discussed similarly. We omit them.

In addition, we mention that the perturbation method in non-Hermitian quantum mechanics can only work effectively when the main part and the perturbation part of a Hamiltonian have the same 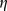-pseudo Hermiticity. However, this requirement is not usually satisfied, such as in our noncommutative model 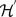 (see eq. (63)) when the second order correction terms are dealt with as perturbation. Therefore, it is not convenient to apply the perturbation method for non-Hermitian Hamiltonian systems. As a whole, we may say it is a more fundamental method to realize the diagonalization for a coupled non-Hermitian Hamiltonian.

At the end of this section, it is quite evident that the eigenvalues and eigenfunctions of our noncommutative generalization turn back to their commutative counterparts when the parameters 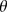 and 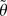 tend to zero. This shows that our noncommutative extension is consistent.

## Conclusion

In this paper, we provide a possible method for a non-Hermitian and non-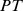-symmetric quantum system. The crucial points of this method are to find out the 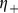 (positive-definite metric) operator and then to define the corresponding annihilation, creation and number operators as in eqs. (5) and (8). After the 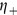-pseudo-Hermiticity that the non-Hermitian and non-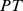-symmetric Hamiltonian inherently possesses is found, the real spectrum is given and the positive-definite inner product, the probability explanation of wave functions, the orthogonality of eigenstates, and the unitarity of time evolution can be confirmed. We apply our method at first to a decoupled system and then to a coupled one by extending the former to the canonical noncommutative space with both noncommutative spatial coordinate operators and noncommutative momentum operators. For the two systems, we find out the specific 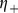 operators and prove the reality of energy spectra and the positive definiteness of inner products, together with the probability explanation of wave functions, the orthogonality of eigenstates, and the unitarity of time evolution. Moreover, to the coupled system we obtain the first and second order corrections of spectra in the noncommutative parameters.

Our results show that it is not mandatory to adopt the biorthonormal basis [6]–[8], [17], [18] for pseudo-Hermitian systems, and that it is still available to use the usual orthonormal basis if the operator 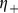 is found. In other words, it is not necessary to introduce 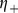 when describing a non-Hermitian Hamiltonian (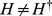) in terms of the biorthonormal basis because the set of eigenstates of 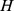 is orthogonal to the set of eigenstates of 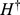. However, in our paper the starting point is to apply the usual orthonormal basis to deal with a non-Hermitian Hamiltonian, thus to introduce 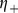 is crucial in order to construct a positive-definite and orthogonal inner product (see eq. (4)) for such a non-Hermitian Hamiltonian. Therefore, we provide a possible method for dealing with non-Hermitian and non-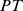-symmetric quantum systems, which is complementary to the 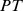-symmetric method.

We note that our two-step method is applicable to the decoupled and coupled Hamiltonians given in the above two sections. We recall that the method based on annihilation and creation operators is not applicable to all coupled (and thus non-diagonalized) Hamiltonians even in the ordinary (Hermitian) quantum mechanics. What we can confirm here is that our method as an earliest attempt from the point of view of annihilation and creation operators is applicable to some coupled and non-diagonalized Hamiltonians in non-Hermitian quantum mechanics. In principle, this two-step method is applicable to other more complex systems, such as a many-body system. The prerequisite is that one has to diagonalize the many-body system at first, and then uses our method. In fact, even in the ordinary quantum mechanics the Fock space approach is usually effective to a diagonalized Hamiltonian system, such as the harmonic oscillator. Our method is a generalization of the Fock space approach to non-Hermitian and non-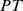-symmetric systems. As a result, for a many-body system governed by a non-Hermitian Hamiltonian, one can still use our two-step method after diagonalizing it.

In addition, we make a comparison between the ordinary (Hermitian) quantum mechanics and the 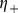-pseudo-Hermitian quantum mechanics. For the former the definitions of the bra and ket vector states, of the inner product, and of the annihilation and creation operators, *etc.*, are model-independent, while for the latter the definitions of the relavent quantities highly depend on the symmetric operator 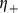 of pseudo-Hermitian systems and thus they are model-dependent since the Hermitian operator 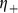 is usually model-dependent. Such a difference has been shown obviously in the present paper. It can easily be understood because the Hermitian quantum systems are the special case when 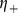 is fixed to be the identity operator from the point of view of 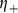-pseudo-Hermitian quantum systems. As a consequence, the former is model-independent because the identity is the only symmetric operator for all Hermitian systems, but the latter is model-dependent because every pseudo-Hermitian system in general has its own symmetric operator 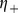. One has to determine the (positive-definite metric) operator 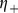 that is in general different from one model to another in pseudo-Hermitian quantum mechanics. It is in principal a hard job to find out an 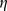 operator for every non-Hermitian and non-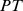-symmetric quantum system, but after all a possible method suggested in this paper is available.

At last, we note that the operator 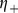 is not unique to a non-Hermitian Hamiltonian. For instance, to the non-Hermitian and non-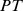-symmetric Hamiltonian we construct in eq. (19), we find another 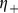: 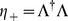, where 
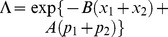
.

Suppose 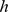 is a Hermitian Hamiltonian that has the same eigenvalues as 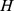 (see eq. (19)), but different eigenfunctions,
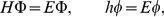
(71)one can introduce an operator 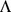, 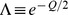, to connect the two representations,
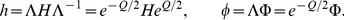
(72)By using the Baker-Campbell-Hausdorff formula,
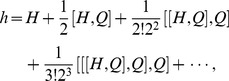
(73)and considering the standard Heisenberg commutation relations of 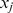 and 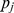, where 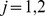, and the Hermiticity of 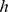, one obtains
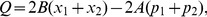
(74)and
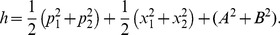
(75)Now using eq. (72) and the Hermiticity of 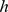, 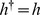, one deduces
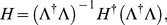
(76)which gives the 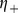-pseudo-Hermiticity of 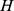, where 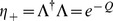.

We note that the orthogonality of eigenstates is ensured in both the Hermitian and 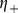-pseudo-Hermitian representations, *i.e.*, if 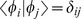 in the Hermitian representation, then we certainly deduce the orthogonality of eigenstates in the 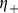-pseudo-Hermitian representation, 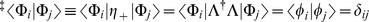.

We can verify that the Hamiltonian eq. (19) satisfies this 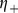-pseudo-Hermitian self-adjoint condition: 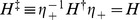. We emphasize that it is only an alternative way for us to give real eigenvalues through the 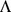 transformation for the non-Hermitian and non-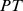-symmetric model. To obtain real eigenvalues is not the sole job for establishing a consistent non-Hermitian quantum theory. In general, one has to consider the other indispensable ingredients, such as the positive definite inner product that relates to the probability explanation of wave functions, the orthogonality of eigenstates, and the unitarity of time evolution, etc. The clarification of the method (see the section of the title **The method for 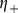-pseudo-Hermitian systems**) of the present paper contains these indispensable ingredients and thus plays an important role in establishing a consistent quantum theory for non-Hermitian Hamiltonians.
